# An autologous blood-derived patch as a hemostatic agent: evidence from thromboelastography experiments and a porcine liver punch biopsy model

**DOI:** 10.1007/s10856-023-06726-2

**Published:** 2023-04-19

**Authors:** Morten P. R. Eskildsen, Otto Kalliokoski, Marie Boennelycke, Rasmus Lundquist, Annette Settnes, Ellen Loekkegaard

**Affiliations:** 1 Department of Obstetrics and Gynecology, Copenhagen University Hospital – North Zealand, Hilleroed, Denmark; 2 Department of Clinical Medicine, University of Copenhagen, Copenhagen, Denmark; 3 Department of Experimental Medicine, University of Copenhagen, Copenhagen, Denmark; 4 Department of Pathology, Copenhagen University Hospital – Rigshospitalet, Copenhagen, Denmark; 5 Reapplix A/S, Birkeroed, Denmark

## Abstract

Perioperative bleeding is a common complication in surgeries that increases morbidity, risk of mortality, and leads to increased socioeconomic costs. In this study we investigated a blood-derived autologous combined leukocyte, platelet, and fibrin patch as a new means of activating coagulation and maintaining hemostasis in a surgical setting. We evaluated the effects of an extract derived from the patch on the clotting of human blood in vitro, using thromboelastography (TEG). The autologous blood-derived patch activated hemostasis, seen as a reduced mean activation time compared to both non-activated controls, kaolin-activated samples, and fibrinogen/thrombin-patch-activated samples. The accelerated clotting was reproducible and did not compromise the quality or stability of the resulting blood clot. We also evaluated the patch in vivo in a porcine liver punch biopsy model. In this surgical model we saw 100% effective hemostasis and a significant reduction of the time-to-hemostasis, when compared to controls. These results were comparable to the hemostatic properties of a commercially available, xenogeneic fibrinogen/thrombin patch. Our findings suggest clinical potential for the autologous blood-derived patch as a hemostatic agent.

**Graphical Abstract:**

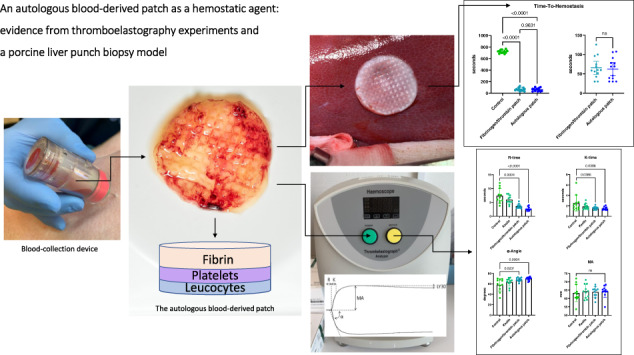

## Introduction

Appropriate hemostasis is essential in any surgery. Bleeding during or after surgery can lead to increases in both morbidity and mortality, longer hospitalization and increased costs to the individual and the society as a whole [1–6]. In addition to conventional surgical interventions, such as suturing and electrocautery, there is a variety of hemostatic agents available [7–11]. These are used as adjunctive or alternatives when the bleeding is diffuse or if hemostasis is difficult to achieve through conventional methods. Most of these agents contain synthetic, animal derived (xenogeneic), or heterologous materials [9]. Consequently, they come with an inherent risk of adverse reactions such as allergies, anaphylaxis, or exaggerated foreign body reactions [12–15]. The use of autologous material could reduce the immunological burden and potentially eliminate some of the harmful side effects to heterologous and xenogeneic materials.

**Fig. 1:**
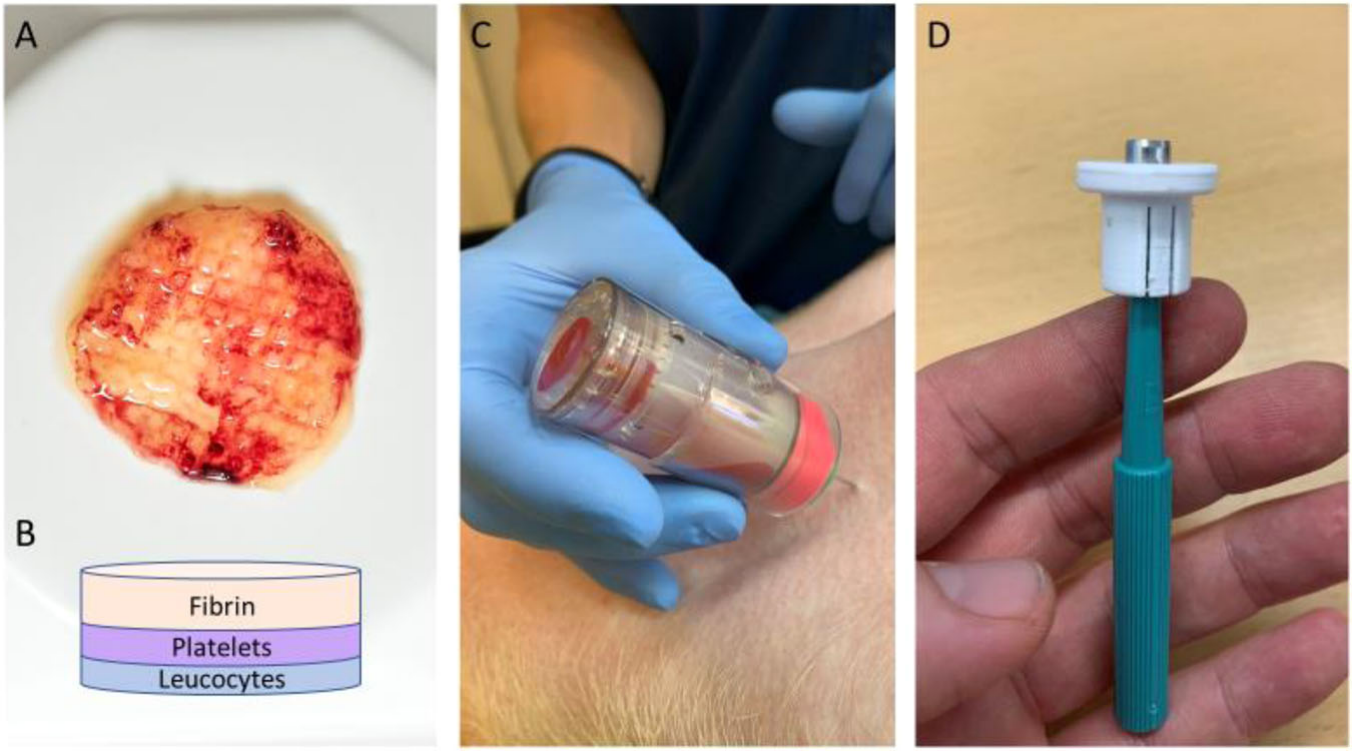
**A** An autologous blood-derived patch. **B** The combined leukocyte, platelet and fibrin structure of the autologous blood-derived patch. **C** 3C Patch® Device used with venipuncture in the jugular vein of a pig. **D** Biopsy punch with stopper mounted

In our group, we have searched for an alternative material for use in surgery. In this context we have worked with an autologous blood-derived patch and have demonstrated that the patch could function as an antiadhesive barrier in a rat model [16]. The patch (Fig. 1A+B) is generated from pure autologous whole blood and consists of three layers: compressed and crosslinked fibrin, thrombocytes, and leucocytes [17]. We did not see any adverse reactions and found the patch to be safe to use inside the abdominal cavity of rats. Additionally, this patch has also proven to be effective in treatment of recalcitrant wounds [18, 19] in humans.

Through pilot studies in both rats and pigs we have observed that the patch has a hemostatic effect in vivo and that the patch can facilitate the clotting process at a bleeding site, for example at a puncture wound in the abdomen. The effect appeared to be similar to that of already existing hemostatic agents [20]. To further explore this apparent hemostatic effect, we wanted to investigate this effect of the patch on blood in vitro. An easily reproduced and standardized analysis of the efficiency of the blood clotting process is thromboelastography (TEG) [21]. This analysis is already implemented and used in the clinical setting [22] where it provides real-time data of the clot formation, clot strength and clot stability of a blood sample at hand. This information comprises the hemostatic status of the blood sample. We therefore wanted to test if the patch was able to accelerate the coagulation process, as seen through thromboelastography. Furthermore, we wanted to test the autologous patch in a relevant animal model, with an organ size and physiological parameters like that of human patients. The pig is well-established as a valuable animal model for translational research [23–25] and has served in multiple studies as models for hemostatic agents [26–32].

In the present study, we hypothesized that the autologous blood-derived patch could function as a topical hemostatic agent. We tested the hemostatic effects of the autologous blood-derived patch both in vitro and in vivo. The autologous blood-derived patch was tested in a parallel with a positive control; a hemostatic xenogeneic patch already with widespread clinical use [20, 33].

## Material and methods

### Ethical considerations

#### Thromboelastography experiment

The experiments relied solely on donated, anonymized human blood from healthy donors. According to Danish legislation, research projects exclusively using anonymous human biological material collected in accordance with the local legislation - that is, material which neither directly nor indirectly can be linked to certain persons - are exempt from approval from the Committee on Health Research Ethics.

#### Animal experiment

The animal experiments were approved by The Animal Experiments Inspectorate under the Danish Ministry of Food, Agriculture and Fisheries (ID: 2020-15-0201-00663). The study was carried out in accordance with the European Union Directive 2010/60/EU for animal experiments. Furthermore, this article adheres to the ARRIVE guidelines (Animal Research: Reporting of In Vivo Experiments). The study protocol was preregistered at https://preclinicaltrials.eu/ (ID: PCTE0000213) prior to commencing the study.

### Hemostatic agents

#### Autologous blood-derived patch

An autologous blood-derived patch (Fig. 1A+B) was made purely from whole blood samples handled in an automated patented process. The resulting patch is a three-layered leukocyte, platelet, and fibrin construct. The patch is currently used in the clinic for hard-to-heal ulcers [18, 19] and has been described earlier in several studies [16, 17, 34, 35].

The process of producing the patch does not differ whether using human or non-human blood. An 18 mL sample of whole blood was drawn. The collection device (3C Patch® device, Reapplix A/S, Denmark, Fig. 1C) is sterile and free of additives like anticoagulants or coagulation activators. The sample was centrifuged (3CP Centrifuge, Reapplix A/S, Denmark) immediately following blood-draw. The length of the procedure is ~12–20 min dependent on coagulation activity. The patches were all produced just prior to commencing the experimental protocol.

#### Xenogeneic fibrinogen/thrombin covered collagen patch

The commercially available xenogeneic fibrinogen/thrombin patch (TachoSil®, Corza Medical GmbH, Germany) has been well described in numerous studies [20, 36] and has been used in surgery in humans since 2004. The patch consists of bovine collagen combined with a layer of heterologous freeze-dried human fibrinogen and thrombin. The patch enhances the natural coagulation process and promotes clotting at the bleeding site [20].

### Thromboelastography model

#### Equipment

A thrombelastograph analyzer (TEG, Haemoscope, Niles, IL 60714-3403, US) with two independent channels was used and connected to a PC with the TEG® Analytical Software (version 4.2.95) installed. The TEG machine was maintained and equilibrated prior to experimenting according to the manufacturer’s protocols.

#### Sample size

In this experiment we wanted to be able to detect a decrease of 25% or more in the reaction time to initiation of clot formation (*R-time)* when comparing a patch-activated sample and a paired non-activated control sample. From a previous study [37] from 2009, the normal value of the *R*-time in whole blood (samples) from 118 healthy adults was reported as 6.8 ± 1.4 min (mean ± SD). We set the alpha level at 5% and decided on 80% statistical power in a paired design, and therefore, ended up with a minimum sample size of eleven.

#### Blood-draw

Eleven healthy anonymous human volunteers donated blood for use in the experiments. All samples were collected by venipuncture by a trained phlebotomist. From each donor, 2 × 5 mL whole blood was drawn into Vacutainer tubes (366575, BD, UK) with 3.2% sodium citrate (0.105 M trisodium citrate), inverted six times, and left for at least 30 min. Furthermore 18 mL of blood was drawn directly into the device, which was used to produce the patch as specified in the section above: “Autologous blood-derived patch”.

#### TEG-analysis

The experiment employed a paired design, each donor acting as their own control. We tested the samples from each donor (*n* = 11) in four different conditions: control (*n* = 11), kaolin-activated (*n* = 11), xenogeneic-fibrinogen/thrombin-patch-activated (*n* = 11), and autologous-blood-derived-patch-activated (*n* = 11).

Samples were kept at room temperature and inverted five times before use. All samples were recalcified before use as described below. For the control samples we used citrated whole blood, which was recalcified with 20 µL calcium chloride (0.2 M CaCl_2_, Haemonetics Corporation, USA) and analyzed without further handling. The kaolin-activated samples were prepared with 1 mL of whole blood added to 40 µL kaolin (Kaolin Reagent, Haemonetics, USA) and mixed by gently inverting five times. The protocol for the patch-activated samples was identical to that of the kaolin-activated. To investigate the effect of the two patches an extract was made from each. This was done as it is not physically possible to add pieces of a patch into the samples. To ensure equal conditions for both patches we standardized the extraction method. A 3 mm punch biopsy of a patch was mixed 15 s with a homogenizer (T10B, IKA, Germany) in 1 mL of PBS (Phosphate Buffered Saline, gibco, UK). We chose PBS, as it has earlier been tested in an in vitro study and did not affect the coagulation in ratios below 4:6 [38]. After mixing, 40 µL of extract was pipetted into a microcentrifuge tube. Subsequently we added 1 mL of whole blood to the extract and mixed it well by inverting the tube five times.

**Fig. 2:**
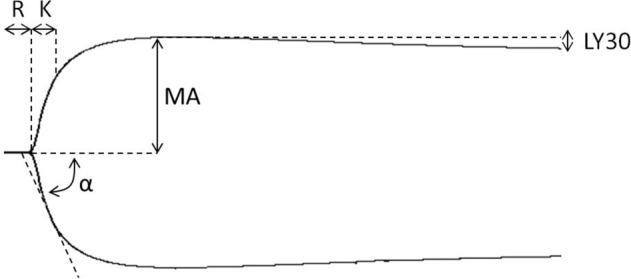
Thromboelastography tracing from one of the donors in the study. R: Reaction time (measured in seconds) - the time from start of test to initial clot formation. K: Kinetics (measured in seconds) – the time to achieve a predefined level of clot strength (amplitude of 20 mm). α: α-angle (measured in degrees) – the speed/rate at which the clot is formed. MA: Maximum amplitude (measured in mm) – the maximum strength of the formed clot. LY30: amplitude at 30 min (measured in % of MA) – the decrease in clot strength at 30 min, representing fibrinolysis

Immediately after recalcification, respectively mixing whole blood with kaolin or extract, 340 µL of the mixture was added to a preheated cup containing 20 µL calcium chloride and the analysis was commenced. All samples were analyzed for two hours. We focused on R-time, *K*-time, α-angle, MA (Maximum Amplitude), and LY30 in all four groups. An example of a thromboelastography-tracing from the experiments with annotations can be seen in Fig. 2. The R-time relates to initial fibrin formation, K-time relates to time of clot formation, α-angle relates to rate of fibrin aggregation and thereby clot formation, maximum amplitude relates to the absolute clot strength and LY30 relates to fibrinolysis and clot stability after 30 min.

### Liver punch biopsy model

#### Animals

A total of thirteen female pigs (*n* = 13, Danish Landrace/Yorkshire, 12 weeks of age and a mean weight of 35 kg on arrival) were enrolled in the hemostasis study. The animals were acclimatized for at least 14 days before being subjected to any procedures. They were housed in groups of four and fed standard pig chow (NAG A.m.b.A., Helsinge, Denmark) twice a day with free access to water. After acclimatizing, the animals entered a surgical procedure related to an adhesion study (to be published separately) before enrolling into the liver punch biopsy model. The adhesion study was an abdominal surgery model where adhesions were created in the lower abdomen between the uterus and the abdominal wall, and the restitution period was 28 ± 1 days. All pigs were in good condition, healthy, and well-recovered from the adhesion study. The mean weight at the enrollment-point of the liver punch biopsy model was 61.1 ± 12.7 kg. None of the pigs had any adhesions in the upper abdomen and their livers appeared macroscopically healthy. The studies were designed to re-use the animals, in accordance with the 3R principles [39, 40] and we judged that the two experiments would not influence one another. The hemostasis study was based on the number of animals available from the adhesion study. This provided us with a sample size of 13 pigs in the study. All of the animals were subjected to the experimental procedure as described below.

#### Experimental procedure (the protocol was adopted and modified from MacDonald et al. [41])

**Fig. 3:**
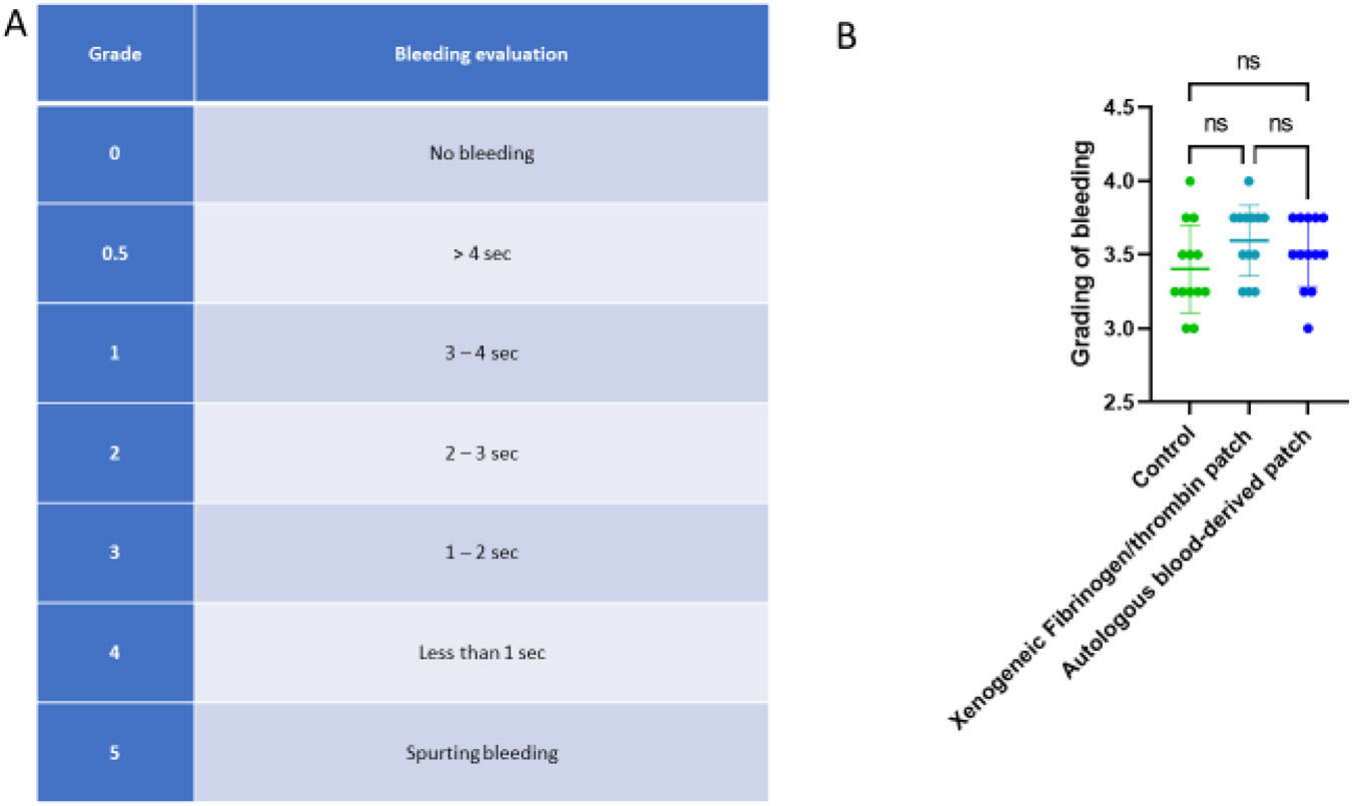
Bleeding scale and comparison of bleedings between groups before treatment. **A** Bleeding scale with grades from 0 to 5. Adapted from Katsuyama et al. [42]. **B** Statistical comparison of bleeding-gradings between groups before treatment. We did not see any significant difference in the level of bleeding-scores between the groups before treatment (mean score ± SD, autologous patch: 3.52 ± 0.24, xenogeneic fibrinogen/thrombin patch: 3.60 ± 0.24, Control: 3.40 ± 0.30. Friedman test: *p* = 0.11). ns not significant

Anesthesia was induced with Zoletil mix 0.14 mL/kg, maintained by Propofol 15 mg/kg/h i.v. with fentanyl 5 µg/kg/h i.v. as analgesic treatment. The animal’s heart rate, oxygen saturation, temperature, and urine output were monitored throughout the surgery. All pigs had a venous blood gas analysis performed before the surgery, after removal of the uterus (see below) and after the completion of the hemostasis protocol, and all received 1000 mL of saline during the surgery. When the animal was sufficiently anesthetized, the surgical procedure was commenced with a ventral abdominal midline incision with a monopolar knife (Valleylab Smoke Evacuation Rocker Switch Pencil, Medtronic, Eindhoven, the Netherlands). The incision was expanded in both cranial and caudal direction, creating a ventral abdominal opening from the pubic bone to the breastbone (*processus xiphoideus*). Hereafter the uterus was removed in toto, as this related to the preceding adhesion study. This, the initial part of the surgery, lasted ~30 min. When hemostasis had been secured, the liver was exposed by creating a chevron-like incision. Space was made for sufficient overview of the lobes and enough to allow for multiple biopsies without readjusting the position of the liver. All other organs and laparotomy edges were covered with gauze and kept moist with saline throughout the surgery. On the diaphragmatic surface of the liver lobes (right lateral lobe, right medial lobe, and left medial lobe) single lesions were created, one at a time, to serve as test sites. With a 6-mm punch biopsy (KAI MEDICAL, Solingen, Germany) mounted with a 3D-printed stopper (Hansenteq ApS, Denmark) (Fig. 1D) we created 3-mm-deep punch biopsies that were grasped with forceps and removed with sharp dissection. The lesions were allowed to bleed freely for a few seconds to obtain a grading. All bleeds were graded according to a predefined score adopted from Katsuyama et al. [42] (Fig. 3). If a bleed was spurting (Grade 5) or not bleeding at all (Grade 0) the lesion was excluded and another lesion created instead, ensuring the same number of lesions per subject. We adopted this score instead of the original score proposed by Macdonald et al. [31, 41, 43] because this score was more representative, as it also contained “No bleeding” and “Spurting bleeding.”

The first bleed in each pig was graded and then treated as an entry control. All experiments were started with an entry control and concluded with an exit control bleed. These controls served to verify that hemostasis could not be obtained within 600 s using only the control protocol. The control-protocol was adopted and modified from MacDonald et al. [41] and served to verify viability of the model. Initially the control bleeding site was compressed with standard cotton gauze for 2 min. Then the compression was lifted. If hemostasis was not observed after 30 s, another 30 s of digital compression was applied. This procedure was repeated for a minimum of 600 s or until hemostasis. Each bleeding site was observed for at least 1 min after the last period of compression that resulted in hemostasis. An entry-control was deemed viable when time-to-hemostasis exceeded 10 min (600 s) after initiation of the control-protocol. All experiments were started with and concluded by a control bleeding, confirming that the model was viable, and the subject could be included in the study. For the study, each time a lesion was created, the hemorrhage was graded according to the predefined score (Fig. 3), as described earlier, and thereafter randomized into one of the following groups: control, autologous blood-derived patch, or xenogeneic fibrinogen/thrombin patch. There were four bleeds in each treatment group (autologous blood-derived patch and xenogeneic fibrinogen/thrombin patch) and four controls, including two designated for entry-bleeding and exit-bleeding each.

The treatments were applied to the lesion and digital compression was applied manually for 2 min. Thereafter the compression was removed, and the lesion was observed, and results recorded. This was the same for both the autologous blood-derive patch group and the xenogeneic fibrinogen/thrombin patch group. The success of the hemostatic device was defined as a cessation of bleeding within 10 min.

Grading of the bleeds was performed by a surgeon in a blinded manner. After the bleed was graded and the lesion randomized to a treatment group (autologous blood-derived patch, xenogeneic fibrinogen/thrombin patch, or control) the surgeon was unblinded to the treatment. As such the grading was blinded and the measurement of time-to-hemostasis was unblinded.

#### Sacrifice of animals

At the end of the study protocol, the pigs were injected with an overdose of Pentobarbital (150–200 mg/kg) while still anesthetized.

#### Evaluation parameters

The bleeding was graded according to a standardized scoring system (Fig. 3).

### Statistical analysis

All statistical testing was performed with R (version i386 4.0.0.).

#### Thromboelastography model

The primary hypothesis was that adding an extract derived from the autologous blood-derived patch would accelerate the coagulation process. We compared the R-time, the K-time, the α-angle, MA, and the LY30 between groups (autologous blood-derived patch versus controls, xenogeneic fibrinogen/thrombin patch versus controls, and autologous blood-derived patch versus xenogeneic fibrinogen/thrombin patch) with Friedman test and subsequently Dunn’s multiple comparisons test with Bonferroni correction.

*P*-values < 0.05 were considered significant.

#### Liver punch biopsy model

The primary hypothesis was that the autologous blood-derived patch and the positive control, the xenogeneic fibrinogen/thrombin patch, would be able to stop bleeding within 600 s and maintain hemostasis thereafter. Gradings of the bleeding sites were compared between groups with a non-parametric Friedman test (autologous blood-derived patch versus xenogeneic fibrinogen/thrombin patch versus controls). Bleeding times were compared within subjects (autologous blood-derived patch versus controls, and xenogeneic fibrinogen/thrombin patch versus controls) with the Wilcoxon matched-pairs signed-rank test. The effects of the autologous blood-derived patch and the positive control was compared with the mean time-to-hemostasis between the two groups (autologous blood-derived patch versus xenogeneic fibrinogen/thrombin patch) using a Wilcoxon matched-pairs signed-rank test. The a priori sample size estimation was based off the related adhesion study. From pilot studies we deemed that this would provide a sufficient sample size to ensure enough power.

*P*-values < 0.05 were considered significant.

## Results

### Thromboelastography experiment

**Fig. 4:**
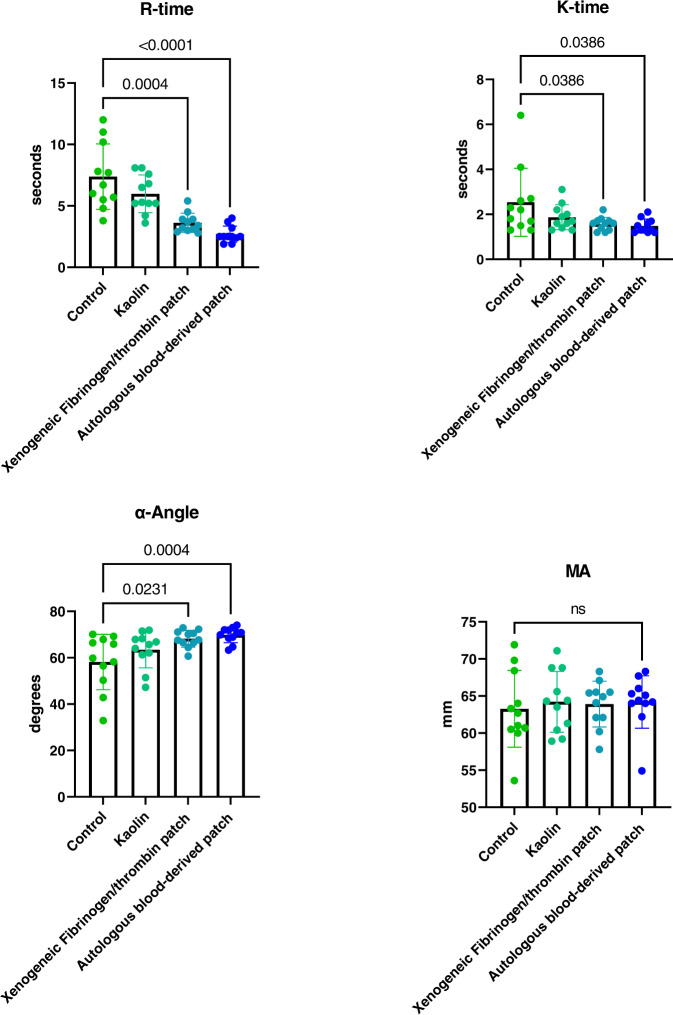
Comparison of thromboelastography results in donated human blood. Both the autologous blood-derived patch and the xenogeneic fibrinogen/thrombin patch decreased the *R*-time and the *K*-time, and significantly increased the α-Angle of the blood when compared to the non-activated blood. There was no significant difference in MA or LY30 (LY30-graph not shown). ns not significant

A total of 11 healthy human volunteers donated blood for use in the thromboelastography experiment. We completed a total of 44 analyses (*n* = 44) distributed in four groups: 11 control samples (recalcified citrated blood only), 11 kaolin-activated samples, 11 fibrinogen/thrombin-patch-activated samples, and 11 autologous blood-derived-patch-activated samples. None of the samples were excluded and all control- and kaolin samples were within the normal range, both as specified by the manufacturer and as reported by a study by Scarpelini et al. [37]. All the data can be seen in Table 1 (showing the *R-time, K-time, α-angle, MA*, and LY30 mean values of all samples) and statistical comparison can be seen in Fig. 4.

**Table 1:** Thromboelastography measurements on whole blood from 11 clinically healthy human donors

Group	R-time (minutes) mean ± SD	K-time (minutes) mean ± SD	α-Angle (degrees) mean ± SD	Maximum amplitude (mm) mean ± SD	LY 30 (%) mean ± SD
Control	7.38 ± 2.66	2.54 ± 1.51	58.18 ± 11.95	63.26 ± 5.18	0.36 ± 0.54
Kaolin	5.98 ± 1.54	1.87 ± 0.56	63.45 ± 7.82	64.21 ± 4.10	0.43 ± 0.50
xenogeneic fibrinogen/thrombin patch	3.62 ± 0.79^a^	1.57 ± 0.29^a^	68.21 ± 3.65^a^	63.91 ± 3.09	0.64 ± 0.64
Autologous blood-derived patch	2.69 ± 0.67^a^	1.48 ± 0.31^a^	69.80 ± 3.32^a^	64.19 ± 3.54	0.50 ± 0.84

Measurements were performed with four different modalities for each donor sample: a control sample, with kaolin-activation, with xenogeneic-fibrinogen/thrombin-patch-activation and with autologous-blood-derived-patch-activation

^a^Significantly different than control, Friedman test with post hoc Dunn’s multiple comparisons test with Bonferroni correction

The mean *R*-time and the mean *K*-time of the autologous-blood-derived-activated samples were significantly lower when compared to controls, but not significantly different from the xenogeneic-fibrinogen/thrombin-patch-activated samples. Mean *R*-time (mean ± SD): autologous blood-derived patch: 2.69 ± 0.67 min, xenogeneic fibrinogen/thrombin patch: 3.62 ± 0.79 min, control: 7.38 ± 2.66 min. Friedman test: *p* ≤ 0.0001. Post hoc Dunn’s multiple comparisons test with Bonferroni correction – autologous blood-derived patch versus control: *p* ≤ 0.0001, autologous blood-derived *patch* versus xenogeneic fibrinogen/thrombin patch*: p* ≥ 0.9999. Mean *K*-time (mean ± SD): autologous blood-derived patch: 1.48 ± 0.31 min, xenogeneic fibrinogen/thrombin patch: 1.57 ± 0.29 min, control: 2.54 ± 1.51 min. Friedman test: *p* = 0.01. Post hoc Dunn’s multiple comparisons test with Bonferroni correction – autologous blood-derived patch versus control: *p* = 0.04, autologous blood-derived *patch* versus xenogeneic fibrinogen/thrombin patch*: p* ≥ 0.9999. Furthermore, we did see a significantly steeper *α*-angle in the samples activated with the autologous blood-derived patch and the xenogeneic fibrinogen/thrombin-activated samples when comparing with the control group, but no difference between the two patches. Mean degrees ± SD: autologous blood-derived patch: 69.8° ± 3.3°, xenogeneic fibrinogen/thrombin patch: 68.2° ± 3.7°, control: 58.2° ± 12.0°, Friedman test: *p* = 0.0005. Post hoc Dunn’s multiple comparisons test with Bonferroni correction – autologous blood-derived patch versus control: *p* = 0.0004, autologous blood-derived *patch* versus xenogeneic fibrinogen/thrombin patch*: p* ≥ 0.9999. We did not find a significant effect of adding the extract from the autologous blood-derived patch or the xenogeneic fibrinogen/thrombin patch extract on MA (*p* = 0.896, Friedman test), or LY30 (*p* = 0.151, Friedman test) when compared to the control samples.

### Liver punch biopsy experiment

**Fig. 5:**
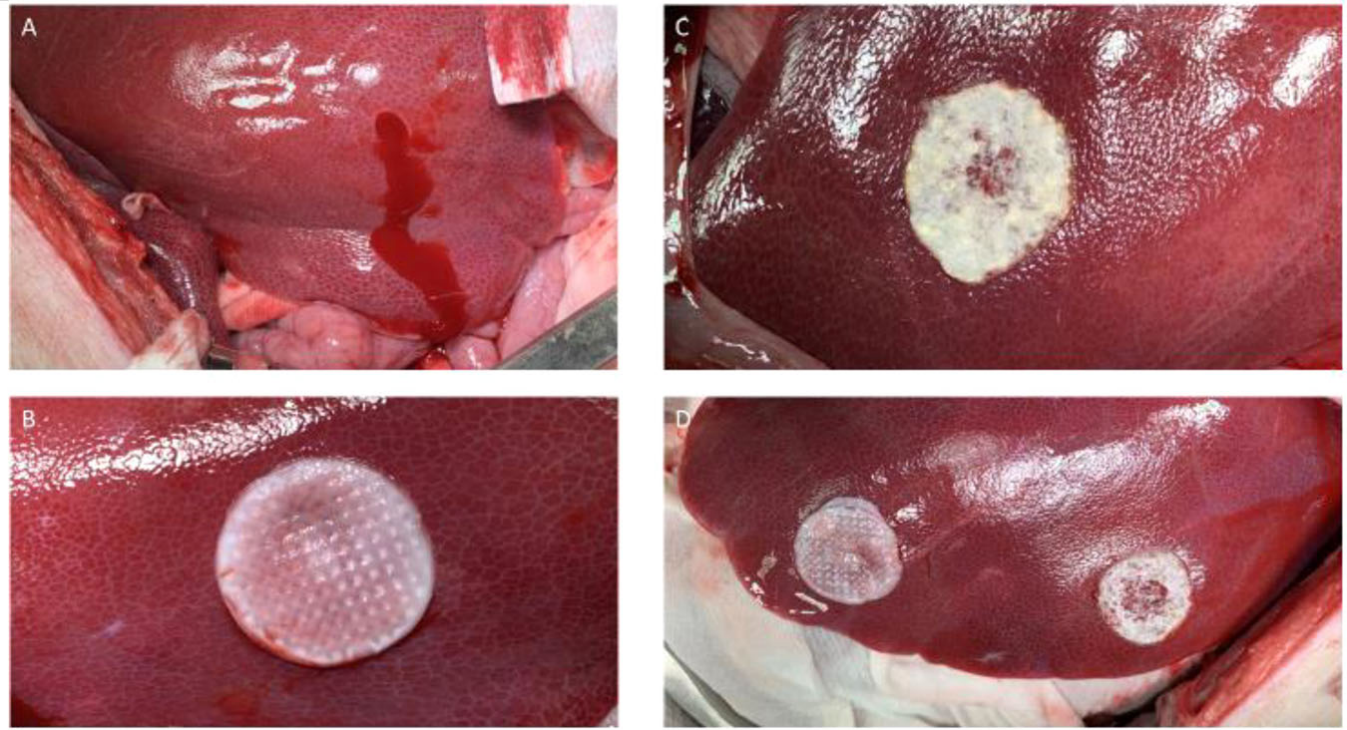
Macroscopic photos from the in vivo porcine liver punch biopsy model. **A** Liver bleeding from a biopsy punch lesion without treatment. **B** Bleeding from a liver punch biopsy wound treated effectively with the autologous blood-derived patch. **C** Bleeding from a liver punch biopsy wound effectively treated with the xenogeneic fibrinogen/thrombin patch. **D** An example of the use of an autologous blood-derived patch and a xenogeneic fibrinogen/thrombin patch on bleedings from a liver punch biopsy wounds in the same animal

A total of 13 pigs successfully completed the hemostasis research protocol. There were no apparent adverse reactions to either of the patches. None of the bleeds were uncontrollable and no pigs were euthanized prematurely. The mean production time to obtain an autologous blood-derived patch was just above 12 min (mean ± SD: 734 ± 123 s, *n* = 13, not including blood sampling). Both the whole blood and xenogeneic fibrinogen/thrombin patches were 100% effective in producing hemostasis within 10 min. None of the bleeds were excluded due to ineffective hemostasis, and all patch-treated bleeds had a time-to-hemostasis below 5 min (300 s). All the 52 bleeds in the control group had a time-to-hemostasis greater than 10 min (mean ± SD: 720.7 ± 28.4 s). This confirmed that the model was indeed appropriate for testing hemostatic agents. Macroscopic photos of the bleeds and treatment with the patches can be seen in Fig. 5.

In 13 pigs we created a total of 177 lesions. Of these, 21 lesions were excluded - 8 were classified as not bleeding and 13 were classified as spurting. The gradings of the lesions did not differ significantly between the autologous blood-derived patch group, the xenogeneic fibrinogen/thrombin patch group, and the control group (mean score ± SD, autologous patch: 3.52 ± 0.24, xenogeneic fibrinogen/thrombin patch: 3.60 ± 0.24, Control: 3.40 ± 0.30. Friedman test: *p* = 0.11), as seen in Fig. 3.

**Fig. 6:**
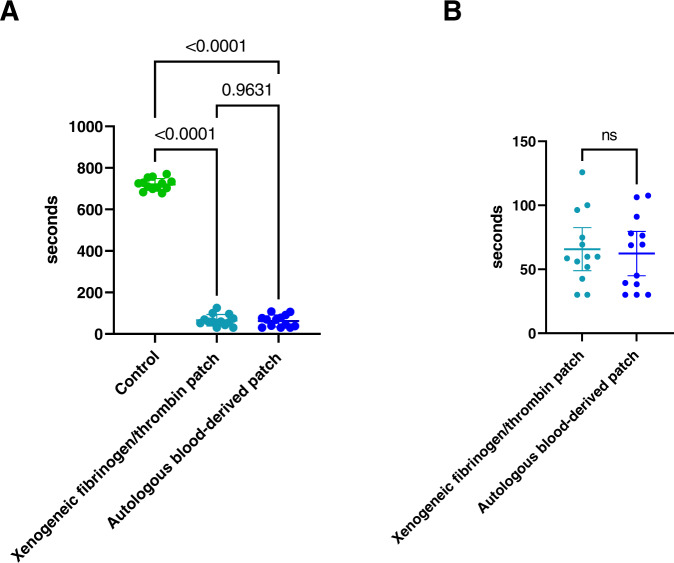
Statistical comparison of time-to-hemostasis between groups from the porcine liver punch biopsy model. **A** Comparison of time-to-hemostasis between the autologous blood-derived patch, the xenogeneic fibrinogen/thrombin patch, and the paired controls. Statistical analysis showed that time-to-hemostasis significantly decreased with both the autologous blood-derived patch and the xenogeneic fibrinogen/thrombin patch when compared to paired controls (mean time-to-hemostasis ± SD; autologous blood-derived patch: 62.26 ± 28.68 s, xenogeneic fibrinogen/thrombin patch: 65.71 ± 27.85 s, controls: 720.77 ± 28.26 s). **B** We did not find a significant difference in the time to hemostasis between the two patches. ns not significant

The autologous blood-derived patch significantly reduced bleeding with a mean time-to-hemostasis of 62.26 ± 28.68 s versus 720.77 ± 28.26 s for the paired control (*p* = 0.0002, Wilcoxon matched-pairs signed rank test, Fig. 6). The mean time-to-hemostasis was also significantly reduced for the xenogeneic fibrinogen/thrombin patch with a mean time-to-hemostasis of 65.71 ± 27.85 s versus 720.77 ± 28.26 s for the paired control (*p* = 0.0002, Wilcoxon matched-pairs signed rank test, Fig. 6). When comparing the effect of the autologous blood-derived patch and the xenogeneic fibrinogen/thrombin patch, we did not find a significant difference in mean time-to-hemostasis (mean ± SD: 62.26 ± 28.68 sec versus 65.71 ± 27.85 s, *p* = 0.48, Wilcoxon matched-pairs signed rank test, Fig. 6).

## Discussion

One of the most common perioperative complications is bleeding. The need for hemostatic agents is obvious and they are used in more than 30% of all surgical procedures [8]. Through thromboelastography and a porcine liver punch biopsy model we have explored an autologous blood-derived patch as a topical hemostatic agent.

In the thromboelastography experiments we observed that adding an extract derived from the autologous blood-derived patch to an autologous blood sample resulted in a significant shortening of the time for the blood to initiate fibrin formation (*R*-time, Fig. 2), which is the starting point for clot formation, compared to both paired controls and kaolin-activated samples. This suggests that the autologous blood-derived patch holds or effectively activates coagulations factors. R-time relates to the coagulation factors [44], and since the autologous patch is derived from whole blood it is reasonable to believe that it contains most of these. Adding the extract did also significantly change the *K*-time, which is the time needed for the clot to reach a predefined clot strength. We also found that the extract from the autologous blood-derived patch was able to significantly increase the α-angle, i.e., the speed at which fibrin builds up in the clot and thereby the rate of clot formation. Finally, our experiments revealed that the activated clots obtained the same maximum strength as their paired controls and that the fibrinolysis at 30 min was not significantly different between groups. In conclusion, these findings demonstrated that the autologous blood-derived patch was able to activate and accelerate the clotting process of whole blood in vitro without compromising the clots build-up, strength, or stability.

In our thromboelastography experiment we also measured the effect of an extract derived from a positive control, a xenogeneic fibrinogen/thrombin patch, a hemostatic agent already approved and used in the clinical setting [45]. The results were largely comparable to that of the autologous blood-derived patch, and we did not find significant differences between the effect of the two extracts from the two patches. The xenogeneic fibrinogen/thrombin patch holds both freeze-dried fibrinogen and freeze-dried thrombin from pooled human plasma [20] and locally activates the last step of the hemostatic cascade, which is conversion of fibrinogen to fibrin [45]. Observing that the extracts derived from the autologous blood-derived patch and the xenogeneic fibrinogen/thrombin patch had similar thromboelastography profiles further supports that clotting factors as for example fibrinogen/thrombin also play a role in the hemostatic effect found using the autologous patch. It is our genuine belief that the thromboelastography experiment demonstrates that the patch holds necessary clotting factors to facilitate clot formation.

We also tested the autologous blood-derived patch as a hemostatic device in a Porcine Liver Punch Biopsy model. The autologous blood-derived patch was able to create hemostasis in 100% of the bleeding sites (hemostatic success was defined as hemostasis within 10 min). It was significantly better than the control protocol, which had 0% hemostatic success. The xenogeneic fibrinogen/thrombin patch was tested as a positive control in the same experiment under identical conditions and were similarly better than the control protocol. There was no significant difference in time-to-hemostasis between the xenogeneic fibrinogen/thrombin patch and the autologous blood-derived patch. The control protocol provided proof of the validity of the model, demonstrating that the bleed would not cease on its own before, during, or after the experiment.

In reviewing the literature, we found that the punch biopsy model was appropriate for testing the autologous blood-derived patch, as it provides lesions that fit the size of the patch, and produces consistent, reproducible, and comparable bleeds. This was confirmed by a pilot study, where we, in addition to using punch biopsies, developed a device to ensure that the depth of each punch biopsy was the same. We modified the original model described by Macdonald et al. [41] by replacing the non-adherent pad, which was used for compression in the control group, with a standard sterile surgical gauze. Surgical gauze is, after all, what most surgeons would use for compression in an actual surgical setting. For this study we used pigs who had a full laparotomy performed prior to the hemostasis protocol due to a preceding surgical procedure as part of an adhesion study. The surgical procedure consisted of removal of uterus, fallopian tubes and ovaries as well as the surrounding connective tissue. The surgery was performed ensuring a minimum of blood loss, and if there was bleeding, this was treated by conventional techniques, such as suturing, compression, or electrocautery. Altogether, performing these two studies in the same session is, in our opinion, quite similar and very relatable to the clinical setting, where perioperative bleeding happens in connection to a surgical procedure and not due to an experimental set up only investigating a hemostatic device.

To our knowledge this is the first preclinical study of a fully autologous blood-derived patch being used for hemostasis, either in vitro or in vivo. Through in vivo pilot studies, a recently published rat study [16] and this study, we found that the autologous blood-derived patch has limitations regarding the lack of adhesion and need for pre-preparation. In pilot studies and in our rat study [16] we observed that the patch did not adhere inadvertently to the surface of undamaged tissue or metal; however, more importantly there was longer lasting adherence to damaged tissues, and especially to blood. This selective adhesion effect might be positive, acknowledging that inappropriate adhesion could be a problem in cases where the surgeons position the patch through laparoscopy trocars, shift the patch between instruments or need to reposition the hemostatic patch. In our opinion some ability to adhere would be a desirable feature in a hemostatic device. In this liver punch biopsy model, we did not investigate the quality and strength of adhesion of the patch but focused purely on the hemostatic effect. However, none of the patches were displaced from the bleeding sites in any of the surgeries. In our opinion the hemostatic effect of the patch is most important. The patch staying in place after obtaining hemostasis is less important, and the presence of the patch in the abdominal cavity is expected to be innocuous based on a former study [16].

With the equipment used for this study, the preparation time of the autologous blood-derived patch ranged between 12 and 18 min depending on the individual patient’s rate of coagulation. Therefore, we propose that the patch should primarily be used in surgeries where the surgeon beforehand knows that there is a high risk of bleeding and a likely need for hemostatic agents, for example in spine-surgery or liver-resection [8]. Preparing the autologous blood-derived patch beforehand and not when the patient is already bleeding would likely ensure that the patch has already preserved clotting factors from the patient’s own blood. Perioperative bleeding often results in deterioration of the ability to coagulate and consumption of coagulation factors [2]. Furthermore, using autologous material would diminish or even abolish the immunological burden of the hemostatic device itself. In general, it is recommended to leave the smallest amount of foreign material inside the body due to fear of adverse events [46, 47], inflammation and foreign body reactions [47–49], something we have not seen with the autologous blood-derived patch [16]. Preoperative preparation would require ~18 mL blood per patch, drawn from the patient before surgery. We found no literature reporting on the consequences of such a small amount of blood-loss preoperatively. In a study of preoperative autologous blood donation [50] of ~300 mL within 2 days of the surgery, the authors found that there was no increase in postoperative anemia. Therefore, we postulate that a blood draw of a rather small amount (18–72 mL corresponding to 1–4 autologous blood-derived patches) just prior to, or during the initiation of surgery would not be harmful to the patient.

## Conclusion

In this study we tested an autologous blood-derived patch as a hemostatic device both in vitro and in vivo. We found that an extract of the patch was able to accelerate the hemostatic process of whole blood in vitro, demonstrated by thromboelastography, without compromising the stability, strength, and quality of the formed blood clot. The autologous blood-derived patch was also able to reduce and stop bleeding in vivo in a liver punch biopsy model. The effect was similar to a positive control, an approved and widespread used xenogeneic hemostatic patch/device. The results indicate that there is a clinical potential for an autologous approach to hemostatic agents. In summary, we believe that the autologous blood-derived patch has the advantage of providing appropriate hemostasis in the absence of the unwanted side effects and adverse events that can be related to xenogeneic devices.

## Data Availability

The data generated or analyzed in this study is available at: Mendeley Data, V1, doi: 10.17632/8hbdrh2trc.1
